# The Site-Specific Recombination System of the *Escherichia coli* Bacteriophage Φ24_B_

**DOI:** 10.3389/fmicb.2020.578056

**Published:** 2020-10-09

**Authors:** Mohammed Radhi Mohaisen, Alan John McCarthy, Evelien M. Adriaenssens, Heather Elizabeth Allison

**Affiliations:** ^1^Department of Functional and Comparative Genomics, Institute of Integrative Biology, University of Liverpool, Liverpool, United Kingdom; ^2^College of Dentistry, University of Anbar, Ramadi, Iraq; ^3^Quadram Institute Bioscience, Norwich, United Kingdom

**Keywords:** Shiga toxin, site-specific DNA recombination, Stx phage, prophage, prophage integrase, tyrosine recombinase, *attP*, *attB*

## Abstract

Stx bacteriophages are members of the lambdoid group of phages and are responsible for Shiga toxin (Stx) production and the dissemination of Shiga toxin genes (*stx*) across shigatoxigenic *Escherichia coli* (STEC). These toxigenic bacteriophage hosts can cause life-threatening illnesses, and Stx is the virulence determinant responsible for the severe nature of infection with enterohemorrhagic *E. coli*, a subset of pathogenic STEC. Stx phages are temperate, and in the present study, the identification of what is actually required for Stx phage Φ24_B_ and bacterial DNA recombination was tested using both *in vitro* and *in situ* recombination assays. It is well established that phage λ, which underpins most of what we understand about lambdoid phage biology, requires its own encoded phage attachment site (*attP*) of 250 bp, a host-encoded attachment site (*attB*) of 21 bp, and a host-encoded DNA binding protein known as integration host factor (IHF). The assays applied in this study enabled the manipulation of the phage attachment site (*attP*) and the bacterial attachment site (*attB*) sequences and the inclusion or exclusion of a host-encoded accessory element known as integration host factor. We were able to demonstrate that the minimal *attP* sequence required by Φ24_B_ phage is between 350 and 427 bp. Unlike phage λ, the minimal necessary flanking sequences for the *attB* site do not appear to be equal in size, with a total length between 62 and 93 bp. Furthermore, we identified that the Φ24_B_ integrase does not require IHF to drive the integration and the recombination process. Understanding how this unusual Stx phage integrase works may enable exploitation of its promiscuous nature in the context of genetic engineering.

## Introduction

Shigatoxigenic *Escherichia coli* (STEC) emerged in the early 1980s as the causal agents of a variety of clinical symptoms and sequelae ranging from mild diarrhea to life-threatening conditions such as hemolytic uremic syndrome and thrombotic thrombocytopenic purpura (Paton and Paton, 1998; Fogg et al., 2011). The key virulence factor of STEC is the expression of Shiga toxin (Stx), which is an AB_5_ toxin encoded on a small operon comprising two genes. The *stx* genes are acquired following an infection with a Stx phage (O’Brien et al., 1984), a temperate lambdoid phage that carries *stx* genes within the late gene region of its genome (Unkmeir and Schmidt, 2000; Smith et al., 2012). Stx phages are members of the lambdoid group of phages which have a common genomic organization and content. The behavior of the archetypal member of this group, phage λ, underpins most of what we understand about lambdoid phage biology. The expression of the *stx* genes is linked to the bacteriophage’s lytic replication cycle, which subverts the host cell’s resources in order to produce more phage particles (Unkmeir and Schmidt, 2000; Wagner et al., 2002; Allison, 2007). However, not all bacteriophage infections result in the lysis of the host cell. DNA from most temperate phages, including phage λ and Stx phages, can, alternatively, become integrated in the host genome, becoming a prophage (Madsen et al., 1999; Saunders et al., 2001) or, in a few other phages, surviving in the cell as a replicating plasmid (Łobocka et al., 2004). For those phage destined to become integrated, the incoming phage produces a site-specific recombinase enzyme that directs phage recombination within the bacterial genome. For lambdoid phages, this recombinase enzyme is known as integrase (Groth and Calos, 2004).

The integrase from phage λ (Int_λ_) is a tyrosine, site-specific recombinase enzyme that drives the recombination between two specific, complementary DNA sequences, the *attP*_λ_ (250 bp) site (Hsu et al., 1980; Mizuuchi and Mizuuchi, 1980; Biswas et al., 2005) located on the phage genome and the *attB*_λ_ site (21 bp) (Mizuuchi and Mizuuchi, 1985) located within the bacterial genome (Mizuuchi and Mizuuchi, 1980; Fogg et al., 2014). The *attP*_λ_ is composed of two integrase binding sites (P and P′) and the core binding site (COC′). The *attB*_λ_ site comprises a central overlap region, known also as O (7 bp), which is flanked by imperfect, inverted repeats, B (7 bp), and B′ (7 bp) (Mizuuchi and Mizuuchi, 1985). Though Int_λ_ is the only phage-encoded protein needed for phage λ integration within the host genome (Fogg et al., 2011), most tyrosine integrases do not act autonomously and require the help of a bacteria-encoded accessory factor (Groth and Calos, 2004). The host-encoded accessory factor required by Int_λ_ is integration host factor (IHF), which works as an accessory protein, enhancing the enzymatic activity of Int_λ_. IHF, a ∼21.8 kDa heterodimeric DNA-binding protein, is composed of two monomers: α (∼11 kDa) and β (∼9.5 kDa) (Kikuchi and Nash, 1978; Azam and Ishihama, 1999; Sanyal et al., 2014), encoded by *himA* and *himD* genes, respectively (Yang and Nash, 1995). IHF interacts with three specific DNA sequences surrounding the *attP*_λ_ site in order to maintain a DNA topography that enables the action of the now active homotetrameric Int_λ_ enzyme (Sugimura and Crothers, 2006). IHF binding sites are around 30–35 bp in size and are composed of at least two domains: the 3′ region and the 5′ region. Unlike the 3′ region, the 5′ region is random in most cases. Alignment among IHF binding sequences within an *attL* population library showed that the 3′ region is where IHF binds strongly and specifically due to the presence of a 13-bp consensus sequence (WATCAANNNNTTR). The 5′ sequences are all simply AT rich and lack any obvious sequence patterns (Goodman et al., 1999).

The crossover regions of *attB*_λ_ and *attP*_λ_ interact with each other through sequence homology (Weisberg et al., 1983), aided by protein–DNA interactions between IHF and specific sites in *attP* that lie between COC and P (Frumerie et al., 2008). The Int_λ_, *attB*_λ_, *attP*_λ_, and IHF complex then allows Int_λ_ to sequentially exchange crossover regions of the *attB* and *attP* sites. Int_λ_ possesses a high binding affinity for the P and P′ sites of *attP* and a low binding affinity to the COC′ site of *attP* (Groth and Calos, 2004). Int_λ_’s recombination mechanism takes the form of a tetrad complex of four Int_λ_ monomers, with each monomer binding to the DNA substrate at the same time (Rajeev et al., 2009). The reaction is started by the Int_λ_ N-terminal domain of each monomer bound to P and P′ in *attP*_λ_ (Biswas et al., 2005). The Int_λ_ C-terminal domains (core binding and catalytic domains) recognize and bind to the complementary crossover sequences (COC′ in the *attP* and BOB′ in the *attB)* regions in a square planer formation, promoting DNA recombination (Van Duyne, 2005). One strand is cut by the first two active Int_λ_ monomers at both the *attB and attP* sites. The next step involves DNA strand exchange and a ligation reaction between one strand from each site, reforming the integrity of the DNA strand. This strand exchange generates a Holliday junction (HJ) intermediate (Biswas et al., 2005). The HJ is resolved when the second pair of integrase monomers carry out the second DNA cut followed by the second DNA strand exchange and ligation, completing the integration event (Hsu and Landy, 1984). This recombination description is based on the “branch migration” model (Weisberg et al., 1983). DNA recombination results in the creation of two new, unique hybrid sites, *attL*_λ_ and *attR*_λ_, differing in sequence from either *attB* or *attP* (Stark et al., 1992). Once this reaction has occurred, the expression of the site-specific recombinase is silenced by the action of the λ repressor (Calendar, 2006; Serra-Moreno et al., 2008).

The lambdoid bacteriophage Φ24_B_ is a Shiga toxin-encoding temperate phage currently classified in the species *Escherichia virus 24B*, the genus *Traversvirus*, the subfamily *Sepvirinae*, and the family *Podoviridae*. It has been well characterized with respect to host range (James et al., 2001), host receptor (Smith et al., 2007a), survival studies (Johannessen et al., 2005), comparative genetic composition (Smith et al., 2007b, 2012), induction signals (James et al., 2001; Fogg et al., 2012; Bloch et al., 2015; Licznerska et al., 2016), and DNA replication proficiency (Nejman et al., 2011; Kozłowska et al., 2017). The integrase of Φ24_B_ and its cognate recombination directionality factor (RDF) have been identified and their expression had been characterized (Fogg et al., 2007, 2011, 2012). The Φ24_B_ *int* gene (EF397940.1) encodes a product (Int_Φ24B_) of ∼45 kDa, whose expression is not controlled by the phage repressor but is expressed constitutively (Fogg et al., 2011). This integrase, Int_Φ24B_, belongs to the tyrosine recombinase family (Esposito and Scocca, 1997; Balding et al., 2005; Fogg et al., 2007). Though various integration sites for the Int_Φ24B_ have been identified (Fogg et al., 2007) and it is understood how prophage induction can be controlled with a constitutively expressed integrase and an inducible RDF (Fogg et al., 2011), little is known about the exact sequences and accessory factors outside the integrase and the core crossover sites (Fogg et al., 2007) that are necessary to drive integration. In this study, we establish working protocols for both *in vitro* and *in situ* integration assays to determine the minimal required sequence lengths of both *attP* and *attB* to support integration at the primary integration site in the *E. coli* chromosome. We also investigate the role of the bacteria-encoded integration host factor in prophage integration.

## Results

### Setting Up an *in vitro* Int_Φ24B_ Assay

**FIGURE 1:**
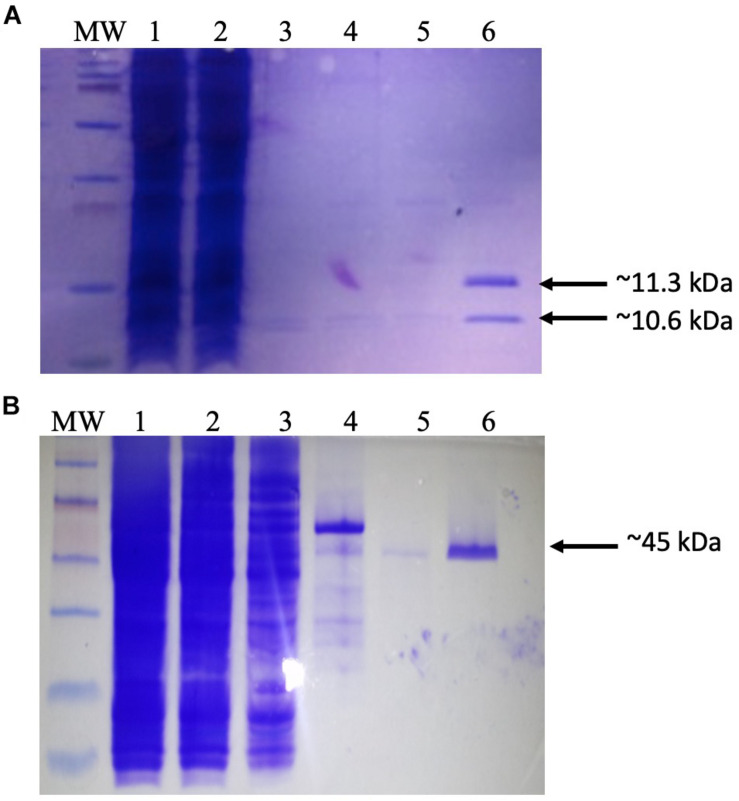
Purification of recombinant proteins. **(A)** Production of dimeric integration host factor (IHF) from the construct pEE2003. *Escherichia coli* strain BL21 carrying the plasmid pEE203 (Supplementary Table 1) was cultured in 10 ml of Luria–Bertani (LB) broth, and overexpression was induced with 5 mM isopropyl β-d-1-thiogalactopyranoside. After 3–4 h, the cells were harvested, and the proteins were purified using a HisTrap (1 ml) column (GE Healthcare Life Sciences). The various steps of this process were analyzed on sodium dodecyl sulfate-polyacrylamide gel electrophoresis (SDS-PAGE) gel. Lanes: MW, prestained molecular weight markers (2–250 kDa) (Bio-Rad); 1, whole-cell lysate; 2, Ni column flow-through; 3–5, column washes; 6, IHF in 60 mM imidazole washing buffer. Arrows indicate the 11.3 kDa his-tagged IhfA subunit and the co-purifying 10.6-kDa IhfB subunit. **(B)** Production and purification of recombinant Int_Φ24B_. *E. coli* strain MC1061 carrying the plasmid pΦ24_B_-int (Supplementary Table 1) was cultured in 10 ml of LB broth, and overexpression was induced with L-arabinose. After 3–4 h, the cells were harvested, and the proteins were purified using a HisTrap (1 ml) column (GE Healthcare Life Sciences). The various steps of this process were analyzed on SDS-PAGE gel. Lanes: MW, pre-stained molecular weight markers (10–250 kDa; Bio-Rad); 1, whole-cell lysate; 2, Ni column flow-through; 3–5, column washes; 6, integrase in 60 mM imidazole. The arrow indicates the purified 45 kDa monomer of Int_Φ24B_.

Φ24_B_ is a member of the lambdoid group of phages based on its genome sequence and genomic context (Smith et al., 2007b, 2012). It was therefore reasonable to assume that Int_Φ24B_ would function in a similar fashion to Int_λ_. To test this hypothesis, an *in vitro* assay was designed. Such an assay required at least four components: purified IHF, purified Int_Φ24B_, and the DNA sequences *attP*_Φ24B_, and *attB*_Φ24B_. The conditions for the induction and the purification of recombinant IHF have been previously published (Frumerie et al., 2005). However, initial attempts to purify the recombinant IHF did not result in the purification of both subunits of the heterodimer. Therefore, the expression plasmid pEE2003 (Frumerie et al., 2005) was recovered and transformed into a different *E. coli* background, strain BL21 (Sambrook et al., 1989), which enabled the purification of both IHF subunits (Figure 1A). Purified recombinant Int_Φ24B_ was obtained from the previously described construct pΦ 24B-int (Fogg et al., 2011), which encodes an active, recombinant integrase with a histidine tag, inducible with arabinose (Figure 1B). Though the exact lengths of the *attB* and *attP* sequences needed to support recombination were unknown, it had been demonstrated that only the *attB* and *attP* sites of 600 bp were sufficient to enable recombination *in situ* (Fogg et al., 2011). Therefore, different lengths of both *attP* and *attB* sequences were produced (Supplementary Tables 1,2) and cloned into pCR2.1 (Supplementary Table 1). The identity of transformants carrying potential *attB* constructs of 100, 200, 300, 400, or 600 bp in length (Fogg et al., 2007) or similarly designed *attP* constructs (100, 200, 300, 400, or 600 bp) was confirmed by PCR using M13 F/R primers, and the introduction of potential point mutations was ruled out by Sanger sequencing.

**FIGURE 2:**
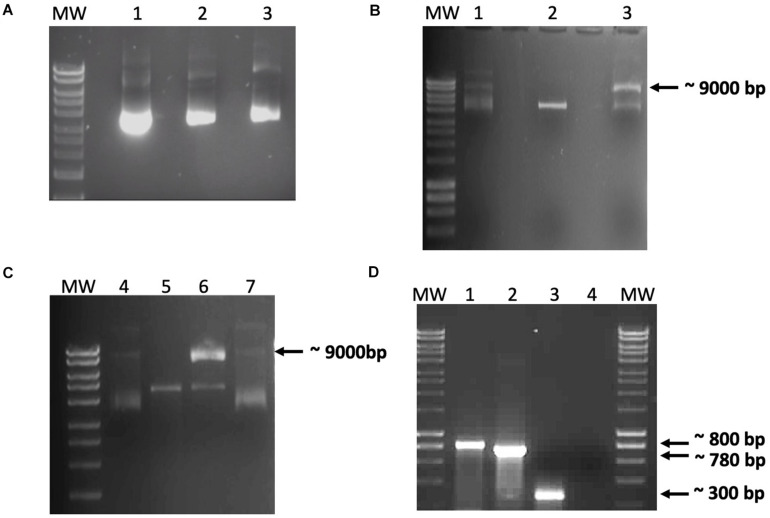
Data from the *in vitro* recombination assay. **(A)** Demonstration that the recombinant integration host factor (IHF) and integrase preparations did not contain nucleases. Purified recombinant IHF and Int_Φ24B_ were incubated with pCR2.1-Φ24_B_-*attP*_600_ to monitor these protein preparations for possible co-purifying nucleases. Lanes: MW, HyperLadder^TM^ 1 kb (Bioline); 1, 100 ng pCR2.1-Φ24_B_-*attP*_600_; 2 and 3, 100 ng pCR2.1-Φ24_B_-*attP*_600_ with 1 μg of IHF or 1 μg of Int_Φ24B_, respectively, incubated at 30°C for 1 h. No co-purifying nuclease activity was detected. **(B,C)** *In vitro* recombination assay results using crude protein extracts. **(B)** Results after incubation for 6 h at 37°C. **(C)** Results after incubation for 24 h at 37°C. Reaction composition by lane numbers: MW, HyperLadder^TM^ 1 kb (Bioline); 1 and 4, supercoiled pCR2.1-Φ24_B_-*attP*_600_ only, without crude protein extract; 2 and 5, linearized pCR2.1-Φ24_B_-*attB*_600_ only, without crude protein extract; 3 and 6, linearized pCR2.1-Φ24_B_-*attB*_600_ and supercoiled pCR2.1-Φ24_B_-*attP*_600_ with 500 ng crude protein extract from TOP10 *E. coli*; 7, supercoiled pCR2.1-Φ24_B_-*attP*_600_ and linearized pCR2.1-Φ24_B_-*attB*_600_ only. Recombination was only detected in lanes 3 and 6 with the presence of a 9-kb band. **(D)** PCR assay of the recombination products. MW, HyperLadder^TM^ 1 kb (Bioline); 1, positive control using M13 F/R primers with pCR2.1-*attP*600 as template; 2, *attRL* PCR fragment amplified using M13 F/R primers; 3, *attRL* PCR fragment amplified using *attP*150 F/attB R primers; 4, negative control using M13 F/R primers without template.

Before mixing the recombinant proteins with the DNA constructs, the absence of nucleases in the recombinant protein preparations was confirmed by incubating either IHF or Int_Φ24B_ with 100 ng of pCR-Φ24_B_-attP_600_ (Figure 2A). The *in vitro* integration assays were then set up to determine if Φ24_B_ integrase was able to promote recombination between *attP* and *attB*. This assay was repeated multiple times with various reagent mixtures using different buffers, with or without the addition of crude cell lysate from *E. coli* TOP10 cells. Recombination events during the *in vitro* assays occurred only under very specific conditions: linearized pCR2.1-Φ 24_B_-attB_600_ and supercoiled pCR2.1-Φ 24_B_-attP_600_ in the presence of Int_Φ24B_ and a crude cell extract of *E. coli* strain TOP10 (Figures 2B,C). The identity of the recombination product was confirmed by PCR using M13 F/R or attRL F/R (attP150F/attBR) oligonucleotide primers (Supplementary Table 2 and Figure 2D), and their identity was verified by Sanger sequencing. It was not clear from these results if the recombinant IHF protein was active or not or whether the assay conditions were not optimized to support recombination activity. Consequently, an *in situ* assay already demonstrated to work reliably (Fogg et al., 2011) was adapted to better characterize the requirements of Int_Φ24B_.

### Characterization of Int_Φ24B_ Requirements Using an *in situ* Assay

**FIGURE 3:**
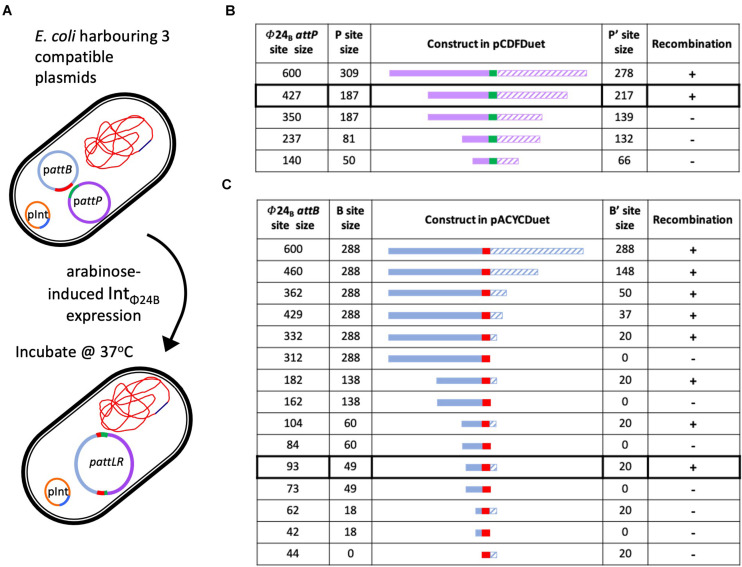
Determining the minimal attP and attB sites utilized by Int_Φ24B_. **(A)** Diagram depicting the *in situ* assay setup, the arabinose induction of Int_Φ24B_ expression, and the resulting chimeric plasmid. **(B)** The design of the various attP constructs (plasmids with nomenclature pΦ24_B_-attP, Supplementary Table 1), providing information on the lengths of the P (solid purple) and P’ (hashed purple) sequences and drawn to scale as they flank the 24 bp central crossover site (green). The detection of recombination (+) or absence (–) is also indicated. **(C)** The design of the various attB constructs (plasmids with nomenclature pΦ24_B_-attB, Supplementary Table 1), providing information on the lengths of the B (solid blue) and B’ (hashed blue) sequences and drawn to scale as they flank the 24-bp central crossover site (red). The detection of recombination (+) or absence (–) is also indicated.

The *in situ* assay required that the cell hosting the recombination events was capable of stable maintenance of three different plasmids (Figure 3A). The plasmid pΦ 24_B_-int replicates with an origin of replication from pBR322, which was also the same origin of replication supporting the replication of the plasmids carrying *attB* and *attP* sites (Supplementary Table 1) used in the *in vitro* recombination assay. To avoid plasmid incompatibility issues in a single *E. coli* cell, all *attB* sequence variants were subcloned into pACYCDuet^TM^-1 (Figure 3C), which carries a p15A origin of replication. All *attP* sequence variants were subcloned into pCDFDuet^TM^-1 (Figure 3B), which carries an origin of replication unrelated to the other two plasmids (Supplementary Table 1). The appropriate plasmids were transformed into *E. coli* TOP10 cells (Supplementary Table 1), Int_Φ24B_ expression was induced with arabinose, and integration events were identified by determining the existence of the large hybrid pΦ24B-*attRL* plasmid (Figure 3A). The integration products were all confirmed by Sanger sequencing. The experiments were run with two replicates. The minimum *attP* sequence necessary to support phage genome integration inside the *E. coli* genome comprised between 350 and 427 bp, with almost equal integrase binding sites (P and P′) site lengths (Figure 3B).

The sequences required by Int_Φ24B_ for activity with regards to *attB* were studied in a similar fashion. The large hybrid pΦ24B-*attRL* plasmid products (Figure 3A) were identified, indicating *attP* and *attB* recombination. The minimal *attB* sequence that could support phage integration comprised between 93 and 62 bp (Figure 3C). The product identities were confirmed by Sanger sequencing. The *attB* site utilized by Int_Φ24B_ is much longer than the 21-bp *attB*_λ_ site utilized by Int_λ_ (Mizuuchi and Mizuuchi, 1985).

### The Role of IHF in Int_Φ24B_ Activity

To test the importance of IHF in promoting the activity of Int_Φ24B_ to drive the integration of its *attP* into the *attB* in the *E. coli* genome, the *E. coli* strain TOP10 used in the *in situ* recombination assay was replaced with the *E. coli* strain JW1702-1 (Baba et al., 2006) in which the *himA* gene has been deleted. A deletion of one of the genes involved in the production of the IHF heterodimer ablates the production of an active IHF. The introduction of pΦ 24_B_-attB_288–288_, pΦ 24_B_-attP_600_, and pΦ 24_B_-int (Supplementary Table 1) into strain JW1702 resulted in the production of a large chimeric DNA product only after the expression of the Int_Φ24B_ was induced by the addition of arabinose (data not shown). This demonstrated that integration could occur in the absence of IHF. However, the requirement of the crude cell lysate in the *in vitro* recombination assay does suggest that another, as of yet unidentified, host-encoded protein is required for Int_Φ24B_ activity.

## Discussion

*Escherichia* phage λ is generally used as a textbook illustration of temperate phage behavior. Our research shows that temperate phage integration, including the presence of host factors and recognition sites, is not generalizable from λ, in particular for the Shiga-toxin encoding phage Φ24_B_. This study sought to better understand how Φ24_B_ mediates integration and to define the factors required for Int_Φ24B_ to function. We know that this integrase exhibits some degree of promiscuity, being able to utilize multiple different sequences in the *E. coli* genome (Fogg et al., 2007), so understanding how this enzyme functions and what factors are necessary for its function could lead to potential biotechnological applications. This knowledge also has important implications for understanding the dissemination of Stx phages and their ability to expand the diversity of Shiga toxin-producing pathogens (Grotiuz et al., 2006; Allison, 2007; Muniesa et al., 2012).

Previous studies have demonstrated that the experimental parameters required for various integrases in *in vitro* recombination assays are not uniform. In the case of recombination catalyzed by the bacteriophage P2-encoded integrase, a tyrosine recombinase, integration requires two *att* sequences, the phage integrase and a bacterial IHF (Sylwan et al., 2010), a phenomenon not uncommon across tyrosine recombinases (Groth and Calos, 2004). However, there are tyrosine recombinases like Cre, encoded by bacteriophage P1, that function without accessory proteins (Groth and Calos, 2004). Due to our lack of controls for the activity of recombinant IHF and Int_Φ24B_, the only interpretable information obtained was that recombination worked best *in vitro* if *attB* was linearized and *attP* was not (Figure 2) and that proteins in a crude cell lysate were essential for recombination. Utilization of a linearized target for phage integration has been described previously, for the integrase encoded by the bacteriophage P2 (Frumerie et al., 2008); however, Int_*P*2_ and Int_Φ24B_ have unrelated integrase box I and box II sites (Balding et al., 2005; Smith et al., 2007b). To better characterize the requirements of Int_Φ24B_, further experiments were run using a proven *in situ* assay (Fogg et al., 2011). The *in situ* assay does not allow the parameters of the recombination events to be strictly controlled in terms of the concentrations and ratios of DNA, protein, enzymes, and reaction buffer content but has been used to reliably detect recombination events (Bliska and Cozzarelli, 1987; McCulloch et al., 1994; Rajeev et al., 2009; Fogg et al., 2011).

Altering our approach to characterize Int_Φ24B_ using an *in situ* assay enabled us to determine the minimal length of the *attP* sequence capable of supporting integration. The P′ side of *attP*_λ_ is ∼80 bp in length, while the P side possesses the integrase binding site and is larger, ∼140 bp (Goodman et al., 1999; Groth and Calos, 2004; Sylwan et al., 2010). A 600-bp DNA fragment harboring the *attP*_Φ24B_ in its center had been shown previously (Fogg et al., 2011) to support integration, so this fragment was used and subsequently shortened to examine the impact of shorter sequence lengths on recombination (Figure 3). In order to examine whether the P and P′ arms flanking the central recombination core need to be of equal sizes or not, *in situ* assays were run using pΦ24_B_-attP_427_, pΦ24_B_-attP_350_, pΦ24_B_-attP_237_, and pΦ24_B_-attP_140_ (Figure 3). The smallest *attP*_Φ24B_ construct supporting recombination was pΦ24_B_-attP_427_. This is considerably larger than the *attP*_λ_ site with *attP*_Φ24B_ P and P′ arm lengths more equal in size.

The *in situ* recombination assay also enabled the determination of minimal *attB* sequence requirements. Tyrosine site-specific recombinases have complex binding sites; however, the *attB* site is usually less complex than the *attP* site. Site *attB*_λ_, for example, is only 21 bp in length, with 7 bp serving as the central crossover core sequence that interacts with the *attP* site (Mizuuchi et al., 1981) and 7 bp on either side serving as the B and B′ arms. Nevertheless, according to our results, *attB*_Φ24B_ site composition is not that simple. The shortest sequence tested that could support recombination was 93 bp long (Figure 3), comprising of a 49-bp B arm followed by the 24-bp crossover site and a 20-bp-long B′ arm. This sequence is very long compared to those of other characterized tyrosine recombinase *attB* sites (Kolot et al., 1999; Lorbach et al., 2000). Some of the larger *attB* sequences characterized for members of the tyrosine recombinase family, e.g., FLP and Cre, require an *attB* sequence ∼50 bp (Abremski and Hoess, 1984; Andrews et al., 1985), while the 62-bp *attB*_Φ24B_ comprising of an 18-bp B arm and a 20-bp B′ arm could not support recombination. The smallest *attB*_Φ24B_ site supports the recombination of an asymmetrical site. This would be very unusual for an *attB* site as most B and B′ arms are of the same size (Kolot et al., 1999; Lorbach et al., 2000), but the resolution of our strategy did not allow us to definitively determine whether the B arm is actually larger than the B′ arm, though it is definitely unusual with regard to its length.

While bacteria-encoded IHF is necessary for the function of most tyrosine site-specific recombinase proteins (Fogg et al., 2014), there are integrases that do not require IHF, e.g., the integrase from the bacteriophage P22 of *Salmonella enterica* serovar Typhimurium can drive recombination in the absence of IHF protein, though IHF can enhance the binding of Int_*P*22_ to *attP*_*P*22_ (Smith-Mungo et al., 1994). Data from two different assays have demonstrated that Int_Φ24B_ functions in the absence of IHF. Firstly, when the assay was performed *in situ* using the JW1702-1 strain of *E. coli* that cannot produce IHF due to the interruption of *himA* (encodes the IhfA subunit), recombination events were detected in an indistinguishable manner from those occurring in the TOP10 *E. coli* background. In line with data from the *in vitro* recombination assay, it became clear that recombinant IHF activity was not required but that some other unknown factor(s) present in the *E. coli* crude protein extract were important. It is highly probable that the reason the *in vitro* assay did not work well under the conditions tested here was not because of the failure to produce biologically active recombinant proteins but was due to the absence of at least one other currently undefined cellular factor/component.

The ability of Int_Φ24B_ to drive phage genome integration inside the *E. coli* cell can have a profound impact on bacterial virulence (Allison, 2007; Fogg et al., 2012). The Stx phage integration results in a cell that, upon clonal expansion, generates numerous progenies that can produce Shiga toxin, the primary virulence factor of EHEC (Paton and Paton, 1998), which are a continuing threat to food safety and public health worldwide. Therefore, it is important to understand how this integrase functions and to identify precisely what is required for it to drive DNA recombination. Each Stx phage characterized to date carries a single integrase gene, and though there are many variants of integrase associated with Stx phages (Smith et al., 2012), Int_Φ24B_ is particularly promiscuous, being able to integrate efficiently into more than one location in the *E. coli* genome (Fogg et al., 2007). Thus far, only the primary *attB_Φ24B_* has been characterized; upon infection, Φ24_B_ attempts to use this site first and will only use a secondary site if the primary site is occupied (Fogg et al., 2007). A comparison here (data not shown) of the now better defined primary *attB_Φ24B_* with the other sequences surrounding the secondary *attB* sites (Fogg et al., 2007) in the *E. coli* chromosome has not revealed any shared homologies beyond those identified by Fogg et al. (2007). More work is needed to identify the *E. coli*-encoded factors used by Int_Φ24B_, how the integrase interacts with the secondary *attB* sites, and what sequences within *attP* and *attB* actually support integrase binding. The work presented here builds upon our knowledge of the DNA sequences required for integration. We have demonstrated that the *attB* sequence needs to be linearized for integration to begin, and we have demonstrated that IHF is not required for Int_Φ24B_-mediated recombination.

## Materials and Methods

### Strains, Plasmids, Media, Buffers, and Oligonucleotides

All bacterial strains and plasmid constructs used in this study are described in appropriate detail in Supplementary Table 1. All oligonucleotide primers used in this study are listed in Supplementary Table 2.

### Protein Overexpression

In order to produce recombinant IHF for the *in vitro* recombination assay, an overnight culture (1 ml) of *E. coli* TOP10 cells (Supplementary Table 1) harboring one of the two histidine-tagged expression constructs, pEE2003 (kindly donated by E. Haggard-Ljungquist) or pΦ24_B_-int, or BL21 cells harboring pEE2003 was used to inoculate 10 ml of Luria–Bertani (LB) agar broth (3.7%; Merck KGaA, Darmstadt, Germany) containing 100 μg/ml ampicillin that was incubated at 37°C with shaking at 200 rpm until the OD_600_ reached 0.5–0.7. A sample (1 ml) was immediately harvested from the culture, and the cells were recovered by centrifugation at 10,000 × *g* for 20–30 min at 4°C and resuspended in 50 μl of Tricine sample buffer [0.1 M Tris–Cl, pH 6.8; 24% (v/v) glycerol; 8% (w/v) SDS, 3.1% (w/v) DTT; 0.02% (w/v) Coomassie Blue R250]. The resuspended cells were frozen at −20°C for later use (non-induced control cells). Expression of the recombinant histidine-tagged protein was induced from the remaining 9 ml of culture using either 0.02% w/v L-arabinose or 5 mM isopropyl β-d-1-thiogalactopyranoside for recombinant histidine-tagged Int_Φ24B_ or IHF, respectively. The induced culture was further incubated at 37°C with shaking at 200 rpm for 3–4 h. The culture was then placed on ice, and a sample (1 ml) was taken. The cells in that 1 ml were harvested by centrifugation at 10,000 × *g* for 20–30 min at 4°C, resuspended in 100 μl of Tricine sample buffer, and frozen at −20°C until needed (induced control). The rest of the cells were harvested by centrifugation at 10,000 × *g* for 20–30 min at 4°C. The resultant pellet was resuspended carefully in 5 ml of ice-cold lysis buffer (50 mM NaH_2_PO_4_, 300 mM NaCl, 10 mM imidazole, pH 8) for purification of the His-tagged protein under native conditions, according to the instructions of QIAGEN’s QIAexpressionist handbook (W. Sussex, United Kingdom). Using a MSE ultrasonic disintegrator (Henderson Biomedical, United Kingdom) with a microtip probe, the sample was treated six to eight times with 10-s pulses at 200–300 W and amplitude of 70 μm. The lysate was kept on ice at all times until it was subjected to centrifugation at 10,000 × *g* for 20–30 min at 4°C. The supernatant was harvested (crude cell extract A, soluble proteins) and stored on ice. The resultant pellet was resuspended in 5 ml lysis buffer (crude extract B, insoluble proteins). Crude extracts A and B (5 μl, each) were mixed with 5 μl of Tricine sample buffer [62.5 mM Tris–HCl, pH 6.8; 2.5% SDS; 0.002% bromophenol blue; 0.7135 M (5%) β-mercaptoethanol; 10% glycerol]. These samples, along with the non-induced and induced control samples, were heated to 95°C for 5 min. The samples were then centrifuged at 15,000 × *g* for 1 min before being subjected to sodium dodecyl sulfate-polyacrylamide gel electrophoresis (SDS-PAGE).

### Protein Purification

Histidine-tagged proteins were purified using the HisTrap (GE Healthcare Life Sciences, Chicago, IL, United States) protein purification 1-ml column utilizing Ni-NTA technology. The column was first washed with water (5 × void volume) before equilibration with an equal volume of lysis buffer. The column was loaded with crude extract A soluble proteins, then washed with several washing buffers (50 mM NaH_2_PO_4_, 300 mM NaCl, pH 8) comprised of varying imidazole concentrations (20, 30, 40, 50, and 60 mM), and finally treated with elution buffer (50 mM NaH_2_PO_4_, 300 mM NaCl, pH 8, with 250 mM imidazole). The volume of the washing buffers and elution buffer used was five times the column void volume. The washes and eluates were collected in graded test tubes. The column was then washed with lysis buffer and water and finally filled with 20% ethanol for storage at 4°C or subsequent reuse. Protein samples were dialyzed, to remove all imidazole, using dialysis tubing (Medicell Membranes Ltd., London, United Kingdom) with a pore size capable of retaining proteins of ≥12–14 kDa and then concentrated using Vivaspin 20 tubes (5 kDa) (GE Healthcare, Buckinghamshire, United Kingdom). The concentration of each protein was measured using the Pierce BCA Protein Assay Kit according to the manufacturer’s instructions. A sample of 15 μl from each elution tube was mixed with 5 μl of Tricine sample buffer, heated to 95°C for 5 min, and loaded onto an SDS-PAGE gel.

### SDS-PAGE

Sodium dodecyl sulfate-polyacrylamide gel electrophoresis (Laemmli, 1970) was performed to examine protein preparations using the Mini-Protean 3 Unit (Bio-Rad, Serial NO 67S). The vertical running gel was 0.75 mm thick and composed of a bottom separating gel (1 ml water, 1.25 ml 3 M Tris–HCl/SDS, pH 8.4, 1.12 ml 40% acrylamide, 0.37 ml glycerol, 5 μl 30% APS, and 5 μl TEMED) and an upper stacking gel (2.03 ml water, 0.77 ml 3 M Tris–HCl/SDS, pH 8.4, 0.31 ml 40% acrylamide, 5 μl 30% APS, and 5 μl TEMED). A 20-μl-sized well-forming comb was used to cover the top of the stacking gel until it was polymerized. The polymerized gel was placed into the electrophoresis tank, with appropriate cathode (0.5 M Tris base, pH 8.45) and anode (1 M Tricine, pH 8.3, with 2% SDS) buffers. Samples (15 μl sample and 5 μl Tricine sample buffer) were loaded in the gel wells using a pre-stained 10–250 kDa molecular weight marker (Bio-Rad) in the outer wells. Electrophoresis was performed at 150 V for 30 min. The unit was dismantled, and the gel was placed in Coomassie blue stain [2% (w/v) Coomassie Blue R250 in 45% (v/v) methanol and 10% (v/v/) glacial acetic acid] with gentle agitation for 30 min. The gel was destained with several exchanges of Coomassie Blue Destain [45% (v/v) methanol and 10% (v/v/) glacial acetic acid] until a clear gel background was obtained.

### Cloning of Bacterial and Phage Attachment Sites in pCR2.1

The *attB* core sequence (Fogg et al., 2007) and either 100, 200 300, 400, or 600 bp of its flanking sequence were amplified from the MC1061 *E. coli* genome using *attB*100 F/R, *attB* 200 F/R, *attB*300 F/R, *attB*400 F/R, or attB 600F/R oligonucleotide primers, respectively.

At the same time, the *attP* core sequence (Fogg et al., 2007) and either 100, 200, 300, 400, or 600 bp of flanking sequence were amplified from the Φ24_B_ genome using *attP*140 F/R, *attP*200 F/R, *attP*300 F/R, *attP*400 F/R, or *attP* 600F/R primers, respectively. The amplification parameters involved an initial denaturation period of 2 min at 95°C followed by 30 cycles of denaturation for 10 s at 95°C, annealing for 10 s at 50°C, extension for 30 s at 72°C, and followed by a final extension period for 2 min at 72°C. The amplification reactions used MyTaq^TM^ (Bioline) DNA polymerase according to the manufacturer’s recommendations. Each of the *attB* or *attP* sequences was cloned, separately, into the pCR2.1 cloning vector (Invitrogen), according to the manufacturer’s guidelines. The *attB* and *attP* pCR2.1 constructs were all individually transformed into chemically competent TOP10 cells. The transformants were cultured overnight on LB agar plates containing ampicillin (100 μg/ml) and kanamycin (50 μg/ml). The identities of the desired transformants were confirmed by colony PCR using primers (*M13 F/R*) flanking the multiple cloning site of the pCR2.1, with the amplification parameters described earlier before they were sent for Sanger sequencing. Freezer stocks were made from each positive transformant and stored at −80°C.

### *In vitro* Recombination Assay

In order to prepare a crude cellular protein extract, cells from an overnight TOP10 culture were harvested by centrifugation for 20–30 min at 10,000 × *g* at 4°C. The resultant pellet was resuspended carefully in 5 ml of ice-cold lysis buffer. The cells were lysed as described above. The lysate was kept on ice at all times, until it was subjected to centrifugation for 20–30 min at 10,000 × *g* at 4°C. Total protein in the supernatant was quantified as described above and used as a crude protein extract.

Recombination assays were always performed in duplicate using either coiled p*attP* plasmid (pCR2.1-Φ24_B_-attP_100_, _200_, _300_, _400_,_or 600_, Supplementary Table 1) with coiled p*attB* plasmid (pCR2.1-Φ24_B_-attB_100_. _200_, _300_, _400_,_or 600_, Supplementary Table 1), coiled p*attP* plasmid with linear p*attB* plasmid, or linear p*attP* plasmid with coiled p*attB* plasmid. Approximately 500 ng of each DNA target species was incubated with or without combinations of recombinant IHF (100 ng, ∼90% purity as determined by SDS-PAGE), recombinant Int_Φ24B_ (200 ng, 90% purity as determined by SDS-PAGE), and/or *E. coli*-derived crude protein extracts (500 ng protein) in a final reaction volume of 50 μl. pCR2.1 plasmids harboring p*attP* or p*attB* sequences were linearized by the restriction enzyme *Bam*HI. Two types of buffer were utilized in these reactions: buffer 1 [20 mM Tris, pH 7.5, 100 mM NaCl, 1% glycerol, and 0.1 mM EDTA (Thorpe and Smith, 1998)] or buffer 2 [50 mM Tris–Cl, pH 7.8, 60 mM KCl, 250 μg of BSA, 0.5 mM EDTA, and 10% glycerol 5 mM spermidine in a final reaction volume of 15 μl (Goodman et al., 1999)]. The reactions were incubated for 6 or 24 h at 37°C and stopped by separating the resulting DNA molecules by Tris-acetate-EDTA (TAE) agarose gel electrophoresis. Any band of the expected recombinant size was excised from the gel, extracted using an Isolate PCR and Gel kit from Bioline, and used as PCR template. If recombination occurred, a unique *attRL* product could be amplified using attRL F/R primers and the amplification parameters described above. The identity of the amplified product was confirmed by Sanger sequencing (GATC Biotech AG, Germany).

### Subcloning *attB* and *attP* Sites Into Compatible Plasmids

The plasmid construct, pCR-Φ 24_B_-*attP*_600_, harboring the *attP* core and 600 bp of flanking sequence was purified from *E. coli* TOP10 strain. The *attP* fragment was recovered using *Bam*HI and *Xho*I restriction endonuclease enzymes. The same pair of enzymes was used to cut the high copy number plasmid, pCDF-Duet. After endonuclease inactivation, by incubation at 65°C for 20 min, the digestion products were each purified on TAE agarose gel and recovered using the Isolate PCR and Gel kit (Bioline, London, United Kingdom). The *attP* sequence was ligated into pCDF-Duet to form pΦ 24_B_-*attP*_600_) before it was transformed into competent TOP10 *E. coli* cells. In parallel, pCR-Φ 24_B_-attB_600_ harboring the *attB* core and 600 bp of flanking sequence, and the medium copy number pACYC-Duet, a plasmid compatible with pCDF-Duet, were also digested using the same endonuclease enzymes (*Bam*HI and *Xho*I), and the products were cloned together as described above to produce pΦ 24_B_-attB_600_ before it was transformed into fresh competent TOP10 cells. This cloning strategy was used repeatedly to produce constructs harboring variously sized *attB* and *attP* sequences.

### *In situ* Recombination Assay

All *in situ* recombination assays were performed inside One Shot TOP10 *E coli* cells (Supplementary Table 1). Each required plasmid transformed sequentially into these cells until all required compatible plasmids for each assay were in one *E. coli* host cell. An appropriate amount of LB broth, supplemented with the plasmid’s resistance antibiotics at their specific concentrations, was inoculated with 1 ml of an overnight culture of cells harboring the compatible plasmids. This culture was incubated at 37°C with shaking at 200 rpm until OD_600_ 0.5–0.8 was reached. Expression of the integrase protein (Int) was induced by the addition of L-arabinose to a final concentration of 0.02% w/v. The cultures were incubated for an additional 3 h at the same incubation temperature. Finally, from each assay flask, 5 ml samples were taken, and all plasmid DNAs present were extracted from the cells in the samples. Recombination events during the *in situ* recombination assays were only determined to have occurred when the following three criteria were met: (1) a PCR reaction using *att*RL F/R primers resulted in the production of DNA product, (2) TAE agarose gel electrophoresis verified the size of the amplification product, and (3) Sanger sequencing of the PCR products confirmed the identity of the hybrid site. All these assays were performed along with a negative control comprised of a sample that was not induced with arabinose so that no Int was produced.

## Data Availability Statement

All datasets presented in this study are included in the article/Supplementary Material.

## Author Contributions

HA and AM conceived the project, oversaw its execution, and contributed to problem solving. MM performed all of the experiments described in the manuscript and made the unique constructs described therein. EA assisted in experimental design and execution. All authors contributed to the article and approved the submitted version.

## Conflict of Interest

The authors declare that the research was conducted in the absence of any commercial or financial relationships that could be construed as a potential conflict of interest.

## Supplementary Material

Supplementary Table 1 Bacterial strains and plasmids.

Supplementary Table 2 Oligonucleotide primers used for PCR amplification of DNA.
