# Supplementary material for: The Site-Specific Recombination System of the Escherichia coli Bacteriophage Φ24B
Source: Front Microbiol. 2020 Oct 9;11:578056. doi: 10.3389/fmicb.2020.578056 (PMC7581858; doi:10.3389/fmicb.2020.578056)
Supplement: Supplementary Table 1 — Bacterial strains and plasmids. [file Table_1.DOCX]

| **Supplementary Table 1. Bacterial strains and plasmids.** | |  |  |
| --- | --- | --- | --- |
| **Bacterial strains** **& plasmids** | **Description** | **Source** | |
| **Bacterial strains** | | | |
| MC1061 | E. coli K-12 derivative, supports lytic and lysogenic infection of Φ24_B_ | (James et al., 2001; Casadaban and Cohen, 1980) | |
| TOP10 | Competent cells, recA-^*^ | Invitrogen | |
| BL21(AI) | araB^∂^::T7RNAP^ℵ^-tetA^℘^, recA- | Invitrogen | |
| JW1702-1 | ΔihfA786::kan^ℑ^ | Coli Genetic Stock Centre (Baba et al., 2006) | |
| **Plasmids** | | | |
| pEE2003 | *Derivative of pET16b, Am^R ♈^ N terminal His6 epitope^ℵ^ fused to himA and untagged himD^∏^ (co-purifies with His-IHF A)* | (Frumerie et al., 2005) | |
| pBAD/Myc-His C | araBAD, C-myc ^∴^/His6 epitope, pBR322 ori^Σ^, Am^R^ | Invitrogen | |
| pCR2.1 | TA Cloning, KanR^⊗^/ Am^R^ | Invitrogen | |
| p$\Phi$24_B_-int | pBAD carrying His6-24_B_ int | (Fogg et al., 2011) | |
| pACYCDuet^TM^-1 | P15A ori^⇑^, Cm^R∃^ | Novagen | |
| pCDF Duet^TM^-1 | CDF ori^ς^, Sp/Sm^R∇^ | Novagen | |
| pCR2.1-$\Phi$24_B_-attB_600_ | pCR2.1 carrying $\Phi$24_B_ attB_1_ (600 bp) | This study | |
| pCR2.1-$\Phi$24_B_-attB_400_ | pCR2.1 carrying $\Phi$24_B_ attB_1_ (400 bp) | This study | |
| pCR2.1-$\Phi$24_B_-attB_300_ | pCR2.1 carrying $\Phi$24_B_ attB_1_ (300 bp) | This study | |
| pCR2.1-$\Phi$24_B_-attB_200_ | pCR2.1 carrying $\Phi$24_B_ attB_1_ (200 bp) | This study | |
| pCR2.1-$\Phi$24_B_-attB_100_ | pCR2.1 carrying $\Phi$24_B_ attB_1_ (100 bp) | This study | |
| pCR2.1-$\Phi$24_B_-attP_600_ | pCR2.1 carrying $\Phi$24_B_ attP (600 bp) | This study | |
| pCR2.1-$\Phi$24_B_-attP_400_ | pCR2.1 carrying $\Phi$24_B_ attP (400 bp) | This study | |
| pCR2.1-$\Phi$24_B_-attP_300_ | pCR2.1 carrying $\Phi$24_B_ attP (300 bp) | This study | |
| pCR2.1-$\Phi$24_B_-attP_200_ | pCR2.1 carrying $\Phi$24_B_ attP (200 bp) | This study | |
| pCR2.1-$\Phi$24_B_-attP_100_ | pCR2.1 carrying $\Phi$24_B_ attP (100 bp) | This study | |
| p$\Phi$24_B_-attB_288-288_ | *pACYCDuet carrying* $\Phi$*24_B_ attB_1_ (600 bp): 288 bp B sequence^$^*, *core sequence,* *and 288 bp B’ sequence^¢^* | This study | |
| p$\Phi$24_B_-attB_138-148_ | pACYCDuet carrying $\Phi$24_B_ attB_1_ (310 bp): 138 bp B sequence, core sequence, and 148 bp B’ sequence | This study | |
| p$\Phi$24_B_-attB_60-50_ | pACYCDuet carrying $\Phi$24_B_ attB_1_ (134 bp): 60 bp B sequence, core sequence, and 50 bp B’ sequence | This study | |
| p$\Phi$24_B_-attB_49-37_ | pACYCDuet carrying $\Phi$24_B_ attB_1_ (110 bp): 49 bp B sequence, core sequence, and 37 bp B’ sequence | This study | |
| p$\Phi$24_B_-attB_49-20_ | pACYCDuet carrying $\Phi$24_B_ attB_1_ (93 bp): 49 bp B sequence, core sequence, and 20 bp B’ sequence | This study | |
| p$\Phi$24_B_-attB_18-20_ | pACYCDuet carrying $\Phi$24_B_ attB_1_ (62 bp):18 bp B sequence, core sequence, and 20 bp B’ sequence | This study | |
| p$\Phi$24_B_-attB_1-0_ | pACYCDuet carrying $\Phi$24_B_ attB_1_ (25 bp): 1 bp B sequence, core sequence, and no B’ sequence | This study | |
| p$\Phi$24_B_-attB_288-148_ | pACYCDuet carrying $\Phi$24_B_ attB_1_ (460 bp): 288 bp B sequence, core sequence, and 148 bp B’ sequence | This study | |
| p$\Phi$24_B_-attB_288-50_ | pACYCDuet carrying $\Phi$24_B_ attB_1_ (362 bp):288 bp B sequence, core sequence, and 50 bp B’ sequence | This study | |
| p$\Phi$24_B_-attB_288-37_ | pACYCDuet carrying $\Phi$24_B_ attB_1_ (349 bp):288 bp B sequence, core sequence, and 37 bp B’ sequence | This study | |
| p$\Phi$24_B_-attB_288-20_ | pACYCDuet carrying $\Phi$24_B_ attB_1_ (332 bp):288 bp B sequence, core sequence, and 20 bp B’ sequence | This study | |
| p$\Phi$24_B_-attB_288-0_ | pACYCDuet carrying $\Phi$24_B_ attB_1_ (312bp): 288 bp B sequence, core sequence, and no B’ sequence | This study | |
| p$\Phi$24_B_-attB_138-20_ | pACYCDuet carrying $\Phi$24_B_ attB_1_ (182 bp): 138 bp B sequence, core sequence, and 20 bp B’ sequence | This study | |
| p$\Phi$24_B_-attB_138-0_ | pACYCDuet carrying $\Phi$24_B_ attB_1_ (162 bp): 138 bp B sequence, core sequence, and no B’ sequence | This study | |
| p$\Phi$24_B_-attB_60-20_ | pACYCDuet carrying $\Phi$24_B_ attB_1_ (104 bp):60 bp B sequence, core sequence, and 20 bp B’ sequence | This study | |
| p$\Phi$24_B_-attB_60-0_ | pACYCDuet carrying $\Phi$24_B_ attB_1_ (84 bp):60 bp B sequence, core sequence no B’ sequence | This study | |
| p$\Phi$24_B_-attB_49-0_ | pACYCDuet carrying $\Phi$24_B_ attB_1_ (73 bp):49 bp B sequence, core sequence, and no B’ sequence | This study | |
| p$\Phi$24_B_-attB_18-0_ | pACYCDuet carrying $\Phi$24_B_ attB_1_ (42 bp):18 bp B sequence, core sequence, and no B’sequence | This study | |
| p$\Phi$24_B_-attB_0-20_ | pACYCDuet carrying $\Phi$24_B_ attB_1_ (44 bp):no B sequence, core sequence, and 20 bp B’sequence | This study | |
| p$\Phi$24_B_-attP_600_ | pCDFDuet carrying $\Phi$24_B_ attP (600 bp): *308 bp P sequence, core sequence, and 278 bp P’ sequence* | This study | |
| p$\Phi$24_B_-attP_427_ | pCDFDuet carrying $\Phi$24_B_ attP (427 bp): *187 bp P sequence, core sequence, and 217 bp P’ sequence* | This study | |
| p$\Phi$24_B_-attP_350_ | pCDFDuet carrying $\Phi$24_B_ attP (350 bp):187 bp P sequence, core sequence, and 139 bp P’ sequence | This study | |
| p$\Phi$24_B_-attP_237_ | pCDFDuet carrying $\Phi$24_B_ attP (237 bp):81 bp P sequence, core sequence, and 132 bp P’ sequence | This study | |
| p$\Phi$24_B_-attP_140_ | pCDFDuet carrying $\Phi$24_B_ attP (140 bp): *50 bp P sequence, core sequence, and 66 bp P’ sequence* | This study | |
| * recA^-^: mutation in DNA repair recombinase gene.^♈^ Am^R^ : Ampicillin resistance marker^∂^ araBAD: arabinose inducible promoter^ℵ^His6: hexa-histidine tag *^$^ sequences: B core binding site*  *^¢^ sequences: B’ core binding site* ^∴^ C-myc: C-terminal myc human oncogene peptide epitope^⊗^ Kan^R^: kanamycin resistance marker^⇑^ P15A ori: P15A replicon.^∃^ Cm^R^ : chloramphenicol resistance marker^ς^ CDF ori: CloDF13-derived CDF replicon^∇^ Sp^R^/Sm^R^: spectinomycin resistance marker^Σ^ pBR332 ori: the pBR322-derived ColE1 replicon *^∏^ himA and himD encode the two subunits of the IHF heterodimer* | | | |
