# Supplementary material for: The Site-Specific Recombination System of the Escherichia coli Bacteriophage Φ24B
Source: Front Microbiol. 2020 Oct 9;11:578056. doi: 10.3389/fmicb.2020.578056 (PMC7581858; doi:10.3389/fmicb.2020.578056)
Supplement: Supplementary Table 2 — Oligonucleotide primers used for PCR amplification of DNA. [file Table_2.DOCX]

| Table 2. Oligonucleotide primers used for PCR amplification of DNA | | |
| --- | --- | --- |
| **Primers** | **Sequence (5’-3’)** | **Tm (**$\boldsymbol{^{\circ}}$**C)** |
| *T7 promoter* | TAATACGACTCACTATAGGG | 60 |
| *T7 terminator* | GCTAGTTATTGCTCAGCGG | 60 |
| *M13 F* | GTAAAACGACGGCCAGT | 60 |
| *M13 R* | CAGGAAACAGCTATGAC | 60 |
| *pBAD F* | ATGCCATAGCATTTTTATCC | 60 |
| *pBAD R* | GATTTAATCTGTATCAGG | 60 |
| *Int F* | TTATATCCATTTAACTAAG | 50 |
| *Int R* | TTAACCGAGCTGTTTA | 50 |
| *Ihf A F* | GCAGAGCGGCCTTTTTA | 65 |
| *Ihf A R* | TCCAGGCATCATTGAGGGA | 65 |
| *ACYCDuetUP1 F* | GGATCTCGACGCTCTCCCT | 65 |
| *pET Upstream* | ATGCGTCCGGCGTAGA | 65 |
| *attP600 F* | CTGAAGGATTTGCGTAAACAGC | 60 |
| *attP600 R* | GATAGTTCCTGCTGGAGTAATCC | 60 |
| *attP 427 F* | ACAGTCGGCGATGGTGA | 55 |
| *attP 427 R* | ATCCTGTACACAAGTTTATAA | 55 |
| *attP 350 F* | CGTGGATCGGTTTAA | 50 |
| *attP 350 R* | GCGTACATATTGAG | 50 |
| *attP 237 F* | AGTGGAGTAAAGAACATGC | 60 |
| *attP 237 R* | CGAACTTACACCTTGATTTTA | 60 |
| *attP 140 F* | GGTACTAATATGACGATTAT | 55 |
| *attP 140 R* | GACGGTATTATCAGTCATA | 55 |
| *attB 600 F* | ATCTGCTTAACGGTGAGCAT | 65 |
| *attB 600 R* | TTAACGGTACGCTGCACTGG | 65 |
| *attB 324 F* | GCGTAAAGCAGAAG | 50 |
| *attB 324 R* | CCATGACGAACTGT | 50 |
| *attB 125 F /*  *attB 110 F* | CCCCGCACTCCATT | 55 |
| *attB 125 R* | GCGAACTGATAAATGG | 55 |
| *attB 110 R* | GTGTCCCCTGCAGGA | 60 |
